# Change in Headache Suffering and Predictors of Headache after Mild Traumatic Brain Injury: A Population-Based, Controlled, Longitudinal Study with Twelve-Month Follow-Up

**DOI:** 10.1089/neu.2018.6328

**Published:** 2019-11-11

**Authors:** Lena H. Nordhaug, Mattias Linde, Turid Follestad, Øystein Njølstad Skandsen, Vera Vik Bjarkø, Toril Skandsen, Anne Vik

**Affiliations:** ^1^ Department of Neuromedicine and Movement Science, Faculty of Medicine and Health Sciences, Norwegian University of Science and Technology, Trondheim, Norway.; ^2^ Department of Public Health and Nursing, Faculty of Medicine and Health Sciences, Norwegian University of Science and Technology, Trondheim, Norway.; ^3^ Department of Physical Medicine and Rehabilitation, St. Olav's Hospital, Trondheim University Hospital, Trondheim, Norway.; ^4^ Department of Neurosurgery, St. Olav's Hospital, Trondheim University Hospital, Trondheim, Norway.; ^5^ Division of Medicine, Stavanger University Hospital, Stavanger, Norway.

**Keywords:** epidemiology, headache, mild traumatic brain injury, MTBI, post-traumatic headache, PTH

## Abstract

Headache attributed to traumatic injury to the head (HAIH) is claimed to be the most common sequela following mild traumatic brain injury (MTBI), but epidemiological evidence is scarce. We explored whether patients with MTBI had an increase in headache suffering following injury compared with controls. We also studied predictors of headache.

The Trondheim MTBI follow-up study is a population-based, controlled, longitudinal study. We recruited patients exposed to MTBI and controls with minor orthopedic injuries from a trauma center and a municipal outpatient clinic, and community controls from the surrounding population. Information on headache was collected through questionnaires at baseline, and 3 and 12 months post-injury. We used a generalized linear mixed model to investigate the development of headache over time in the three groups, and logistic regression to identify predictors of headache.

We included 378 patients exposed to MTBI, 82 trauma controls, and 83 community controls. The MTBI-group had a larger increase in odds of headache from baseline to the first 3 months post-injury than the controls, but not from baseline to 3–12 months post-injury. Predictors for acute HAIH were female sex and pathological imaging findings on computed tomography (CT) or magnetic resonance imaging (MRI). Predictors for persistent HAIH were prior MTBI, being injured under the influence of alcohol, and acute HAIH. Patients who experience HAIH during the first 3 months post-injury have a good chance to improve before 12 months post-injury. Female sex, imaging findings on CT or MRI, prior MTBI, and being injured under the influence of alcohol may predict exacerbation of headache.

## Introduction

Headache is claimed to be one of the most frequent symptoms following mild traumatic brain injury (MTBI), manifested both as new onset and worsening of pre-existing headache.^1,2^ The *International Classification of Headache Disorders* (3rd edition, ICHD-3) defines headache attributed to traumatic injury to the head (HAIH) as a headache with no defining clinical characteristics that starts within 7 days of injury.^1^ Acute HAIH is of shorter, and persistent HAIH is of greater duration than 3 months.^1^

Because headache is a common disorder, research on HAIH is especially challenging. The head injury population are not headache-free pre-injury, but rather a part of the general population, of which in Norway 37% report having suffered from headache during the last year.^3^ In studies with lack of controls or biased selection it is therefore difficult to distinguish a pre-existing headache from a newly formed HAIH.^2,4^ It is vital to include a control group for comparison.^5,6^ The majority of studies published on HAIH are based on cases from hospital admissions or special clinics where the participants may be highly selected with regard to symptoms.^4^ Hospitalization of patients with MTBI has become less common, and a population-based design including both non-hospitalized and hospitalized patients is therefore important for a study population without selection bias.^7^

The incidence of MTBI is high, and a close follow-up of all patients would require a large effort by the health care service.^8^ To identify patients in need of intervention it is important to recognize those at risk for developing HAIH. Previous studies have identified several predictors, such as sex, age, multiple head injuries, and a number of acute symptoms following the head injury.^2,9–13^ However, there is large variation in follow-up time and poor consistency between study results.^14^

The main aim of this prospective, controlled study, including both hospitalized and non-hospitalized patients with MTBI, was to explore if patients with MTBI had an increase in headache suffering after the injury compared with a control group with minor orthopedic injuries and a community control group. The second aim was to study predictors of acute and persistent HAIH in the MTBI group.

## Methods

### Study design and study population

This population-based, controlled, prospective cohort study included three study groups: one group exposed to MTBI, one group exposed to minor orthopedic injuries but no head injury (trauma controls), and one group of community controls not exposed to any injury. The MTBI group and the trauma controls were recruited from two emergency departments (EDs) in Trondheim, Norway: St. Olav's Hospital (Trondheim University Hospital), a regional Level 1 trauma center, and Trondheim Municipal Emergency Clinic, a general practitioner-run, outpatient clinic, with a catchment area of 229,000 inhabitants. The community control group was recruited from the same catchment area and matched to the MTBI group with regard to age, sex, and education. The trauma control group was matched with regard to age and sex.

### Inclusion in the Trondheim MTBI follow-up study

Details of how the MTBI group was recruited, diagnostic criteria, and exclusion criteria have been described earlier.^15^ The inclusion period was April 2014 to December 2015 for the MTBI group and June 2014 to December 2017 for the controls. Inclusion criteria for the MTBI group were having experienced MTBI and age 16–59 years. TBI was categorized as mild according to World Health Organization (WHO) criteria: Glasgow Coma Scale (GCS) score 13–15 at presentation, and either witnessed loss of consciousness (LOC) <30 min, confusion, or post-traumatic amnesia (PTA) <24 h.^16^ Inclusion criteria for trauma controls were fractures or symptoms from soft-tissue injuries lasting ≥48 h. Exclusion criteria were the same as for patients with MTBI; also excluded were head or neck injury (including whiplash injury), multi-trauma, and trauma to dominant upper extremity.

### Study variables

Information regarding the injury resulting in an MTBI and clinical symptoms at the ED, including information about alcohol influence at the time of injury, was collected through medical records and by interviewing the participants. The GCS score was observed by the study personnel or recorded from the medical record. If lacking, the history and clinical descriptions in the medical records were used to estimate a score. This method was used on 29 (7.7%) of the MTBI participants. Duration of PTA was recorded as the time after injury for which the patient had no continuous memory (<1 h, or 1–24 h). Previous MTBI was defined as having experienced one or more head injuries fulfilling diagnostic criteria for MTBI. The clinical radiology report was used to classify CT findings into intracranial findings and cranial fractures. An MRI examination was performed on a 3.0 Tesla Siemens Skyra System (3T, susceptibility-weighted imaging [SWI], fluid-attenuated inversion recovery [FLAIR], diffusion-weighted imaging [DWI]) if the patient lived within a 1-h drive, there was a time slot for MRI available within 72 h, and the patient consented to MRI (see Supplementary Appendix S1 for protocol). Intracranial traumatic pathology and cranial fractures on MRI or CT were dichotomized into “imaging findings on CT or MRI” (yes/no).

### Headache variables

**FIG. 1:**
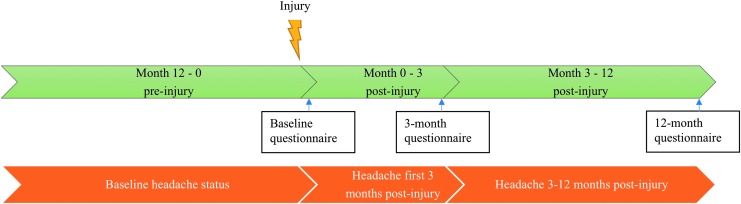
Timeline illustrating the time periods covered in each questionnaire. Baseline questionnaire: headache suffering last 12 months prior to injury (MTBI and trauma controls) or first questionnaire (community controls). Three-month questionnaire: headache suffering the first 3 months post-injury. Twelve-month questionnaire: headache suffering 3- 12 months post-injury. MTBI, mild traumatic brain injury. Color image is available online.

Information regarding the participants' headache status was collected through self-administered questionnaires at three points in time (Fig. 1). In the questionnaire at baseline the participants were asked the headache screening question, “Have you suffered from headache during the last year?”; those who answered “yes” were recorded as suffering from headache at baseline. Participants answering “yes” to the headache screening question answered the subsequent headache questions that enabled classification into definite migraine, probable migraine, and tension-type headache (TTH) according to the ICHD-3 criteria.^1^ The participants answered the same headache questions after 3 and 12 months and were then asked to report headache during the previous 3 and 9 months, respectively.

Participants who answered “no” to the first screening question were considered non-sufferers. Participants were not specifically asked about the duration of untreated headache attacks, because some individuals always use attack medication for their headaches. Because of this, the ICHD-3 criteria for migraine were modified so that duration of <4 h was accepted as well. Chronic migraine was defined as headache >14 days/month without medication overuse and fulfilling criteria for migraine. Chronic TTH was defined as headache >14 days/month without medication overuse and fulfilling criteria for TTH. Chronic daily headache (CDH) was defined as headache occurring >14 days/month. The participants were also asked to report use of acute medication for headache during the previous month, enabling the diagnosis of medication overuse headache (MOH) defined as CDH with acute medication use >14 days/month. The validity of these questionnaire-based diagnoses has previously been evaluated and found to be acceptable.^17^

To be able to examine predictors for new headache or exacerbation of previously reported headache from baseline to the first 3 months post-injury (acute HAIH) and from baseline to 3–12 months post-injury (persistent HAIH) respectively, headache sufferers were classified with regard to headache frequency. Each participant was then categorized into one of the following four groups: no headache suffering, headache suffering <7 days/month, headache suffering 7–14 days/month, and headache suffering >14 days/month. We defined exacerbation of headache as new onset of headache or increased frequency of previously reported headache.

### Other variables

Demographic variables and other health-related variables were collected through an interview with the study participants within 2 weeks after the injury. These were the known confounders: age, sex, socioeconomic status, and AUDIT score (alcohol use identification test), categorized from a continuous variable into two categories with score boundaries <8 and ≥8.^18^ Lower secondary school grades were chosen as measure of socioeconomic status because of the young age of many of the participants, making it difficult to use the traditional measures such as income or duration of education. In Norway, lower secondary school grades have been found to correlate strongly with the socioeconomic status in the family.^19^

### Statistical analysis

Continuous data are presented as means and standard deviations (SDs), whereas categorical data are presented as frequencies and percentages. We used a generalized linear mixed model (GLMM) to compare the development of headache status regarding headache suffering or no headache suffering between the three groups (MTBI, community controls, and trauma controls) over time (baseline, and 3 months and 12 months post-injury). To control for known confounders, the model included age, sex, socioeconomic status, and alcohol use as covariates, in addition to group, time, and a group-time interaction term. A random, subject-specific intercept on the logit scale was included to account for within-subject dependencies. Missing outcome variables for subjects with at least one observation on outcome were handled by the model, whereas missing explanatory variables were handled by listwise deletion.

We used logistic regression to identify predictors for exacerbation of headache within the first 3 months post-injury (acute HAIH) among the participants answering both the baseline and 3-month questionnaires, and exacerbation of headache 3–12 months post-injury (persistent HAIH) among the participants answering both the baseline and 12-month questionnaires, for the MTBI participants. We examined sex (male/female), age (continuous variable), socioeconomic status (continuous variable), GCS score (13–14/15), PTA (1–24 h/<1 h), previous MTBI (yes/no), pathological imaging findings (fracture or intracranial pathology) on CT or MRI (yes/no), and being influenced by alcohol at time of injury (clinically assessed or self-reported) (yes/no) as potential predictors. In addition, we performed a separate analysis examining acute HAIH (yes/no) as a predictor for persistent HAIH, including the previously mentioned predictors as covariates. Missing data were handled by listwise deletion.

Linearity of age as a continuous variable with respect to the logit of the probability of headache (yes/no) was assessed with the Box-Tidwell procedure.^20^ Age was found to be linearly related to the logit of the dependent variable and could thus be included as a continuous variable. The results for both analyses are presented as odds ratios (ORs) with 95% confidence intervals (CIs). *P*-values <0.05 were considered statistically significant. We used IBM SPSS Statistics software version 25 for the logistic regression and Stata/MP version 15.1 for the GLMM.

### Ethical approval and patient consents

The Regional Committee for Research Ethics approved the study (REK 2013/754). Participants, or parents of participants <18 years of age, gave informed consent.

## Results

### Participants

The MTBI group consisted of 378 participants. The control groups consisted of 82 trauma controls and 83 community controls. Questionnaire response rates are reported in Table 1. Injury characteristics for the MTBI group and demographic data for the MTBI group and the two control groups are presented in Table 2.

**Table 1.** Questionnaire Response Rates in All Groups

	*MTBI*	*TC*	*CC*	
	N *(%)*	N *(%)*	N *(%)*	*Total*
Baseline	272 (72.0)	78 (95.1)	76 (91.6)	426 (78.5)
3 months	232 (61.4)	75 (91.5)	41 (49.4)	348 (64.1)
12 months	236 (62.4)	58 (78.4)	67 (80.7)	361 (66.5)

CC, community controls; MTBI, mild traumatic brain injury; TC, trauma controls.

**Table 2.** Demographics and Injury Characteristics

	*MTBI*	*CC*	*TC*
Variable			
Total number of subjects	378	83	82
Age at participation (years) (mean ± SD)	31.2 ± 13.0	33.1 ± 13.0	32.6 ± 13.0
Male	247 (65.3)	49 (59.0)	51 (62.2)
Lower secondary school grades (range: 1-6)^a^			
2.0-2.9	18 (4.8)	2 (2.4)	0
3.0-3.9	99 (26.2)	20 (24.1)	15 (18.3)
4.0-4.9	169 (44.7)	37 (44.6)	45 (54.9)
5.0-6.0	82 (21.7)	22 (26.5)	21 (25.6)
Missing	10 (2.6)	2 (2.4)	1 (1.2)
AUDIT ≥8	108 (40.6)	26 (34.7)	22 (27.8)
Baseline headache suffering	83 (30.5)	29 (38.2)	21 (26.9)
Alcohol influence at time of injury^b^	169 (44.7)	-	6 (7.3)
Admitted to hospital	118 (31.2)	-	11 (13.4)
Injury mechanism			
Fall	135 (35.7)	-	26 (31.7)
Violence	65 (17.2)	-	1 (1.2)
Bicycle	58 (15.3)	-	7 (8.5)
Sport	54 (14.3)	-	30 (36.6)
MVA	43 (11.4)	-	3 (3.7)
Struck by object	17 (4.5)	-	6 (7.3)
Other	3 (0.8)	-	-
Unknown	3 (0.8)	-	-
GCS score		-	-
15	277 (73.2)		
14	57 (15.1)		
13	5 (1.3)		
Missing	39 (10.3)		
PTA			
<1	271 (71.7)	-	-
1-24 h	107 (28.3)	-	-
CT	299 (79.1)	-	-
MRI within 72 h	194 (51.3)	-	-
Imaging findings (CT/MRI)	37 (9.8)		
Cranial fracture (CT/MRI)	16 (4.2)		
Intracranial finding (CT/MRI)	32 (8.5)		
Cranial fracture and intracranial finding (CT/MRI)	11 (2.9)		

^a^ 6 is the highest grade, and 1 is the lowest.

^b^ Based on clinical assessment.

The data are presented as *n* (%) if not otherwise stated.

AUDIT, alcohol use identification test; CI, confidence interval; CC, community controls; CT, computed tomography; GCS, Glasgow Coma Scale; MRI, magnetic resonance imaging; MTBI, mild traumatic brain injury; MVA, motor vehicle accident; OR, odds ratio; PTA, post-traumatic amnesia; SD, standard deviation; TC, trauma controls.

The mean age in the MTBI group was 31.2 years (SD: 13.0 years) and 65.3% were male. Only 31.2% of the MTBI participants were admitted to a hospital; 51.7% of these were observed <24 h.

Traumatic pathology was revealed in 37 (9.8%) of the MTBI participants. Intracranial pathology was revealed in 32 (8.5%), 16 (4.2%) had cranial fractures, and 11 (2.9%) had both cranial fracture and intracranial pathology.

### Headache trajectories in the MTBI group and the two control groups

The proportion reporting baseline headache suffering was 30.5% of the MTBI group, 26.9% of the trauma controls, and 38.2% of the community controls. The odds of baseline headache suffering were not significantly different between the three groups (Table 3). The MTBI group had a higher odds of reporting headache suffering during the first 3 months post-injury than the trauma controls (OR: 6.94; 95% CI: 2.45-19.64), but were not significantly different from the community controls. The odds for headache suffering between the groups 3–12 months post-injury were not significantly different from each other (Table 3).

**Table 3.** Odds Ratios for Headache Suffering between the Groups at Baseline, the First 3 Months Post-Injury and 3-12 Months Post-Injury

	*Baseline*			*First 3 months post-injury*			*3-12 months post-injury*		
	*OR*	*95% CI*	P*-value*	*OR*	*95% CI*	P*-value*	*OR*	*95% CI*	P*-value*
MTBI vs. TC	1.60	0.57-4.51	0.372	6.94	2.45-19.64	<0.001	2.73	0.93-8.05	0.068
MTBI vs. CC	0.76	0.29-1.97	0.567	2.69	0.85-8.58	0.094	0.91	0.33-2.52	0.862
TC vs. CC	0.47	0.15-1.46	0.192	0.39	0.11-1.38	0.144	0.33	0.10-1.10	0.072

Baseline: headache suffering last 12 months prior to injury (MTBI and TC) or first questionnaire (CC).

CC, community controls; CI, confidence interval; MTBI, mild traumatic brain injury; OR, odds ratio; TC, trauma controls.

Table 4 shows that for the MTBI group, the odds of headache increased significantly from baseline to the first 3 months post-injury (OR: 8.61; 95% CI: 4.90-15.13) and from baseline to 3–12 months post-injury (OR: 3.72; 95% CI: 2.19-6.31). There was a significant decrease in odds of headache from the first 3 months post-injury to the next 9 months (OR: 0.43; 95% CI: 0.25-0.73). In the trauma control group there were no significant changes over time. In the community control group there was a significant increase in the odds of headache from baseline to 3–12 months post-injury (OR: 3.07; 95% CI: 1.23-7.65).

**Table 4.** Odds Ratios for Change in Odds of Headache Suffering from Baseline to the First 3 Months Post-Injury and 3-12 Months Post-Injury within Each Group

	*MTBI*			*TC*			*CC*		
	*OR*	*95% CI*	P*-value*	*OR*	*95% CI*	P*-value*	*OR*	*95% CI*	P*-value*
First 3 months post-injury vs. baseline	8.61	4.90-15.13	<0.001	1.98	0.80-4.95	0.140	2.42	0.83-6.99	0.104
3-12 months post-injury vs. baseline	3.72	2.19-6.31	<0.001	2.17	0.81-5.81	0.121	3.07	1.23-7.65	0.016
3-12 months post-injury vs. first 3 months post-injury	0.43	0.25-0.73	0.002	1.09	0.43-2.81	0.851	1.27	0.43-3.74	0.662

Baseline: headache suffering last 12 months prior to injury (MTBI and TC) or first questionnaire (CC).

CC, community controls; CI, confidence interval; MTBI, mild traumatic brain injury; OR, odds ratio; TC, trauma controls.

**FIG. 2.**
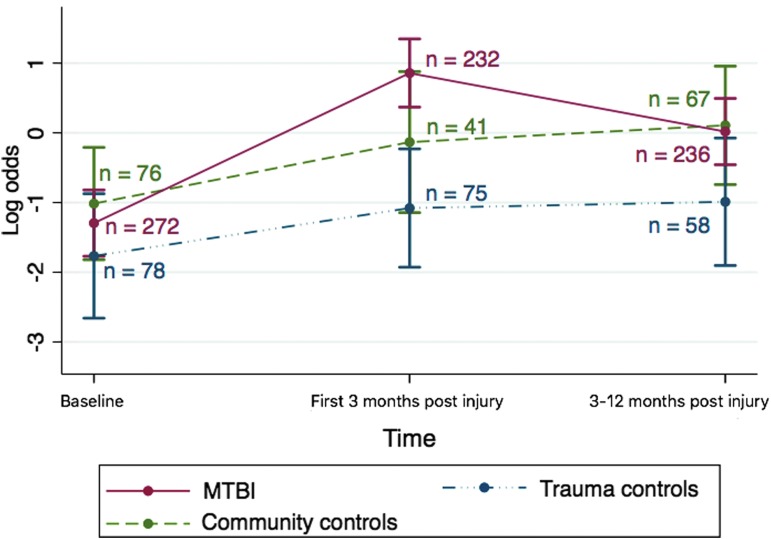
Estimated values of log odds with 95% CI for headache pre-injury, the first 3 months post-injury, and 3–12 months post-injury for the MTBI group and two control groups. Baseline: headache suffering last 12 months prior to injury (MTBI and trauma controls) or first questionnaire (community controls). Three-month questionnaire: headache suffering the first 3 months post-injury. Twelve-month questionnaire: headache suffering 3–12 months post-injury. CI, confidence interval; MTBI, mild traumatic brain injury. Color image is available online.

The development in odds for headache over time differed significantly between the groups (overall *p* = 0.035 for the interaction between time and group). Figure 2 shows the trajectories of log odds for headache in the MTBI group and the two control groups.

Table 5 shows that the increase in odds of headache from baseline to the first 3 months post-injury was significantly larger for the MTBI group than for the trauma controls (ratio of OR: 4.33; 95% CI: 1.50-12.47) and the community controls (ratio of OR: 3.56; 95% CI: 1.09-11.66). The ORs of change in headache from baseline to 3–12 months post-injury did not differ between the groups.

**Table 5.** Ratio of Odds Ratios for the Change in Headache Suffering from Baseline to the First 3 Months Post-Injury and 3-12 Months Post-Injury in the Three Participation Groups Related to Each Other

	*First 3 months post-injury vs. baseline*			*3-12 months post-injury vs. baseline*			*3-12 months post-injury vs. first 3 months post-injury*		
	*Ratio of OR*	*95% CI*	P*-value*	*Ratio of OR*	*95% CI*	P*-value*	*Ratio of OR*	*95% CI*	P*-value*
MTBI vs. TC	4.33	1.50-12.47	0.007	1.71	0.57-5.15	0.342	0.39	0.13-1.17	0.093
MTBI vs. CC	3.56	1.09-11.66	0.036	1.21	0.43-3.42	0.719	0.34	0.10-1-13	0.078
TC vs. CC	0.82	0.20-3.32	0.784	0.71	0.19-2.68	0.612	0.86	0.21-3.60	0.837

Baseline: headache suffering last 12 months prior to injury (MTBI and TC) or first questionnaire (CC).

CC, healthy controls; CI, confidence interval; MTBI, mild traumatic brain injury; OR, odds ratio; TC, trauma controls.

### Exacerbation of headache in the MTBI group

**FIG. 3.**
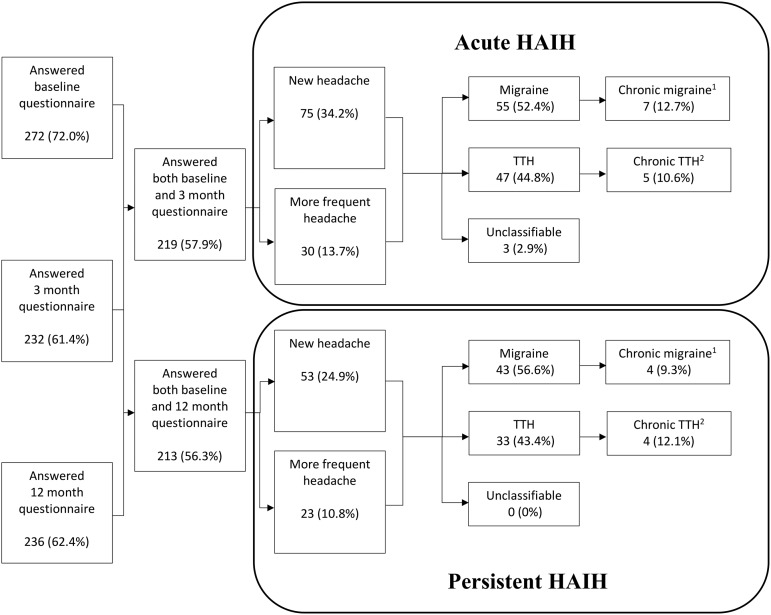
The proportion of participants in the MTBI group who developed new headache or exacerbation of pre-existing headache including headache subgroup. Chronic migraine^1^: headache >14 days/month without medication overuse and fulfilling criteria for migraine. Chronic TTH^2^: headache >14 days/month without medication overuse and fulfilling criteria for TTH. HAIH, headache attributed to traumatic injury to the head; MTBI, mild traumatic brain injury; TTH, tension-type headache.

Figure 3 shows the share of participants in the MTBI group who developed exacerbation of headache, including headache subgroup. When classifying the HAIHs as primary headaches according to ICHD-3, migraine was the most common phenotype. During the first 3 months post-injury, 47.9% of the subjects reported exacerbation of headache. Of these, 49.5% reported migraine headache, 21.9% (*n* = 23) reported CDH, and 8.6% (*n* = 9) reported MOH. During the 3–12-month post-injury period, 35.7% of the subjects reported exacerbation of headache compared with baseline. Of these, 57.9% reported migraine headache, 19.7% (*n* = 15) reported CDH, and 9.2% (*n* = 7) reported MOH.

### Predictors of exacerbation of headache in the MTBI group

Female sex (OR: 2.52; 95% CI: 1.35-4.72) and pathological imaging findings on CT or MRI (OR: 2.88; 95% CI: 1.16-7.15) were significant positive predictors for acute HAIH, but this was no longer the case for persistent HAIH (Table 6). Significant positive predictors for persistent HAIH were prior MTBI (OR: 2.89; 95% CI: 1.28-6.53) and being injured under the influence of alcohol (OR: 2.06; 95% CI: 1.04-4.09). Age, socioeconomic status, duration of PTA, and GCS score were not significant predictors of acute or persistent HAIH. In a follow-up analysis for persistent HAIH, we also included “having acute HAIH” in the regression model. This variable was highly significantly associated with persistent HAIH (OR: 5.63; 95% CI: 2.54-12.50) (not shown in table).

**Table 6.** Predictors of New Headache or Exacerbation of Previously Reported Headache from Baseline to the First 3 Months Post-Injury (Acute HAIH) and 3-12 Months Post-Injury (Persistent HAIH)

	*Acute HAIH*				*Persistent HAIH*			
*Variables*	*N*	*OR*	*95% CI*	P*-value*	*N*	*OR*	*95% CI*	P*-value*
Female	74	2.52	1.35-4.72	0.004	74	1.84	0.97-3.51	0.063
Age (years)	189	0.99	0.97-1.01	0.349	188	0.99	0.97-1.01	0.377
Socioeconomic status^a^	189	1.23	0.85-1.79	0.269	188	1.17	0.79-1.73	0.425
Prior MTBI	39	1.63	0.76-3.49	0.207	34	2.89	1.28-6.53	0.010
Imaging finding^b^ (CT/MRI)	29	2.88	1.16-7.15	0.022	28	0.83	0.33-2.11	0.699
PTA >1 h	51	0.72	0.33-1.57	0.406	50	0.49	0.22-1.13	0.095
GCS <15	33	1.00	0.44-2.30	0.999	36	1.54	0.66-3.61	0.319
Injured under the influence of alcohol^c^	71	0.98	0.49-1.93	0.958	73	2.06	1.04-4.09	0.039

^a^ Lower secondary school grades were used as measure of socioeconomic status.

^b^ A combined variable for intracranial pathology and cranial fractures.

^c^ Based on clinical assessment.

CI, confidence interval; CT, computed tomography; GCS, Glasgow Coma Scale; HAIH, headache attributed to traumatic injury to the head; MRI, magnetic resonance imaging; MTBI, mild traumatic brain injury; OR, odds ratio; PTA, post-traumatic amnesia.

## Discussion

In this large, prospective, controlled study, MTBI participants had a larger increase in odds of headache from baseline to the first 3 months post-injury than both control groups, but the change in odds of headache from baseline to 3–12 months post-injury did not differ between the groups. Predictors for acute HAIH were female sex and pathological imaging findings on CT or MRI. For persistent HAIH the predictors were prior MTBI, being injured under the influence of alcohol, and acute HAIH.

The patients with MTBI had a significant increase in odds of headache between baseline and the first 3 months post-injury and between baseline and 3–12 months post-injury. From the first 3 months post-injury to 3–12 months post-injury they had a decrease in odds of headache. The increase in odds of headache from baseline to the first 3 months post-injury was significantly larger for the MTBI group than for trauma controls and the community controls. However, the ORs of headache from baseline to 3–12 months post-injury did not differ between the groups. We also saw, somewhat surprisingly, that the control groups had an increase in headache suffering from baseline to 3–12 months post-injury, but this was only statistically significant for community controls.

This increase in odds of headache suffering in the control groups, seen together with the decrease in odds of headache suffering from the first 3 months post-injury to 3–12 months post-injury in the MTBI group could explain why the MTBI group did not differ from the control groups between baseline and 3–12 months post-injury. The increase in odds of headache suffering from baseline to 3–12 months post-injury in the control groups could have been caused by participation bias (those who suffer from headache are more likely to continue answering the questionnaires throughout the study); another explanation could be that we had a young study population and many of our study participants were in the age where headache disorders start.^21^ But this could also be a demonstration of the dynamic course of headache. In a study of a random sample of 5,000 adults selected from five general practices in the United Kingdom, the authors showed that 24% of respondents without recent headache (last 3 months) at baseline reported headache in at least one follow-up the following year.^22^ Hence, increase in headache suffering could be observed also in persons not exposed to a head injury.

Taken together, our results showed that the MTBI participants developed acute HAIH. We cannot, however, conclude that the MTBI group developed persistent HAIH, because the control groups without MTBI also had an increase in odds of headache suffering.

Our findings of acute HAIH but not persistent HAIH are in line with a controlled study from Lithuania and a population-based study from Norway.^23,24^ In the Lithuanian study, migraine occurred significantly more often in patients with MTBI than in patients with a minor orthopedic trauma 3 months post-injury, but headache diagnoses and headache frequency occurred in similar proportions as in the controls 12 months post-injury.^23^ The authors of the Norwegian study did not find any association between previous head injury and headache 22 years after hospitalization for head injury.^24^ However, the large difference in follow-up time (12 months vs. 22 years) warrants caution when comparing these results with our findings.

In our study, 47.9% of the participants in the MTBI group reported exacerbation of headache from baseline to the first 3 months post-injury and 35.7% from baseline to 3–12 months post-injury. This is somewhat lower than the findings in a prospective study without controls on hospitalized patients with MTBI from the United States, where 62% of the subjects reported exacerbation of headache at 3 months and 58% at 12 months.^12^ However, only 18% of the participants in the American study reported having a problem with headache pre-injury. This is considerably lower than in the general population in the United States, which gives reason to suspect recall bias.^25^ Another explanation could be that the study included only hospitalized patients, which could mean their population had more severe MTBI than ours.^12^

Headache is more prevalent among women than men in the general population.^3,26^ In our study, female sex was a significant positive predictor of acute HAIH. This is in line with several other studies that have shown female sex to be a risk factor of HAIH.^2,9,10,27,28^

Imaging findings on CT or MRI (intracranial traumatic pathology and/or cranial fractures) were a significant positive predictor of acute HAIH but not persistent HAIH. However, in a recent study from Korea, the authors found that HAIH occurred more frequently 12 months post-injury in patients with minimal traumatic intracranial hemorrhage after MTBI than in those without.^29^ Research on imaging findings as a predictor for headache is scarce, but some studies have investigated imaging findings as a predictor for post-concussive symptoms (PCS), a cluster of post-traumatic symptoms, including headache. A prospective study from Finland found no significant differences in the rates of PCS 1 month and 1 year following MTBI, in participants with or without MRI abnormalities.^30^ A prospective study from the United States found no statistically significant differences in the rates of PCS at 3 or 6 months following MTBI between participants with or without imaging findings on CT or MRI.^31^

Prior MTBI was a significant positive predictor of persistent HAIH in our study. This is consistent with several previous studies. A retrospective study that examined the risk of CDH found that the odds of CDH increased with the number of lifetime head or neck injuries.^32^ In a previous population-based historical cohort study, we found that subjects having more than one head injury showed an even greater odds of headache compared with controls than all individuals exposed to head injury without specification of iteration.^11^ Finally, in a study aiming to predict which patients were at risk for developing PCS (with a median duration of 19 weeks), multiple head injuries was one of the predictors^33^

Being injured under the influence of alcohol was another significant positive predictor of persistent HAIH. We cannot conclude whether being injured under the influence of alcohol is a proxy for a generally high alcohol consumption or if being injured under the influence of alcohol is a true risk factor of developing HAIH. The association between alcohol influence at the time of injury and the damage that occurs in the brain must be examined specifically to answer this question.

Finally, we found that acute HAIH was a significant positive predictor for persistent HAIH. This is in line with the results from a Norwegian study that examined predictors of headache 22 years following head injury.^10^

### Strengths

The major strengths of this study are its population-based and prospective study design and appropriate comparison groups. This study emphasizes the importance of including control groups, as we saw a significant increase in headache suffering also in the community control group. We used a validated headache questionnaire and a renowned MTBI classification that makes the study accessible to comparison with other studies, also future ones.^16,17^ We did a baseline assessment of pre-injury headache suffering, wherein the proportions reporting headache suffering during the last year prior to the MTBI were similar in all groups. The proportions were also very similar to a population-based study on headache prevalence in Trøndelag county in Norway in 2006–08, in which the same validated headache questionnaire as in the present study was used.^3^ This means that our study is at low risk for recall bias. The 12-month follow-up time allowed us to examine persistent HAIH, which according to ICHD-3, is of greater than 3 months' duration.^1^ We included both hospitalized MTBI participants and non-hospitalized MTBI participants from a general practitioner-run, out-patient clinic. We therefore believe that there was low risk of selection bias, which is a problem when the study population is recruited from trauma hospitals, headache clinics, or special clinics with a focus on health problems following TBI. In the statistical analysis, we controlled for known confounders for MTBI and headache, such as age, sex, socioeconomic status, and alcohol use.

### Limitations

In studies where the participants are included after their MTBI, assessment of pre-injury headache suffering may always be influenced by recall problems. Also, participants with more headache suffering could be more likely to continue to answer all questionnaires throughout a study because they have more personal interest in it.^34^ This may, like recall bias, lead to an exaggerated increase in headache suffering throughout the study. This could be the reason for the increase in headache suffering in the community control group. One could speculate that because so many of the trauma controls got their injury during sport activities and fewer were influenced by alcohol at the time of injury, they represent a healthier group than the MTBI group and community controls. We had a young study population and many of our study participants were in the age where headache disorders start.^21^ Also, we did not ask the participants about the time of onset for their exacerbation of headache. Therefore, we cannot specify if the onset of headache was within 7 days after head injury, which is a criterion for classifying a headache as HAIH, according to ICHD-3.^1^ However, the authors of ICHD-3 state that this criterion is somewhat arbitrary and conclude that further research is needed to determine which interval might be more appropriate.^1^Further, the community control group had a low response rate at 3 months (49.4%). Finally, our results may not be valid for all age groups, because we only studied participants from 16 to 59 years of age.

### Implications for public health

A considerable proportion of patients exposed to an MTBI will experience new headache or exacerbation of previously reported headache. However, a large number of these will improve during the first year following the MTBI and this should be the main message to the patients. However, prior MTBI, being injured under the influence of alcohol, and having acute HAIH are positive predictors for persistent HAIH, and these patients should receive greater attention by the health care service. Of the patients exposed to MTBI who developed persistent HAIH, 9.2% fulfilled criteria for MOH. The corresponding proportion in the general population is 1.0%.^3^ Also, previous research has found MOH to be more prevalent among persons exposed to head injury.^11,35^

## Conclusion

Exacerbation of headache during the first 3 months after MTBI is likely related to the head injury, and predictors for acute HAIH were female sex and pathological imaging findings on CT or MR. However, many patients will experience improvement of their headache before reaching 12 months post-injury. Patients exposed to MTBI should be advised against overuse of analgesics, and management of patients with persistent HAIH should include treatment against MOH when medication overuse is suspected.

## Supplementary Material

Supplemental data
