# Supplementary material for: Change in Headache Suffering and Predictors of Headache after Mild Traumatic Brain Injury: A Population-Based, Controlled, Longitudinal Study with Twelve-Month Follow-Up
Source: J Neurotrauma. 2019 Nov 11;36(23):3244–52. doi: 10.1089/neu.2018.6328 (PMC6857461; doi:10.1089/neu.2018.6328)
Supplement: Supplemental data [file Supp_App1.pdf]

Supplementary Data

Supplementary Appendix S1

Description of the MRI protocol

Magnetic resonance imaging (MRI) was performed on a 3.0T Siemens Skyra System (Siemens Healthcare, Erlangen, Germany), software version E11C, with a 32-channel head coil. The image protocol consisted of a series of clinical MRI sequences:

1. 3D T1-weighted magnetization-prepared rapid gradient-echo (MP RAGE)
2. 2D Diffusion weighted imaging (DWI)
3. 3D T2 space
4. 3D T2-weighted fluid attenuated inversion recovery (FLAIR)
5. 3D T2-weighted suceptibility-weighted imaging (SWI)

The intracranial traumatic MRI findings were categorized into: (1) traumatic axonal injury, (2) contusions, (3) epidural hematoma, (4) subdural hematoma, and (5) traumatic subarachnoid hemorrhage.
